# Application of HARM Score to Measure Surgical Quality and Outcomes in Bariatric Patients

**DOI:** 10.1007/s11695-018-3253-5

**Published:** 2018-04-27

**Authors:** Michał R. Janik, Rami R. Mustafa, Tomasz G. Rogula, Adel Alhaj Saleh, Mujjahid Abbas, Leena Khaitan

**Affiliations:** 1 University Hospital Cleveland Medical Center / Case Western Reserve University School of Medicine, Cleveland, OH USA; 2 Department of General, Oncologic, Metabolic and Thoracic Surgery, Military Institute of Medicine, Radziwie 7/370, 01-164 Warszawa, Poland; 3 General Surgery Department, Faculty of Medicine, Menoufia University, Al Minufya, Egypt; 4 Faculty of Medicine and Health Sciences, Jan Kochanowski University, Kielce, Poland; 5 Department of Surgery, Texas Tech University Health Sciences Center, Lubbock, TX USA

**Keywords:** Bariatric surgery, Outcome measurement, Quality indicator, HARM score, Complications, Laparoscopic sleeve gastrectomy, Laparoscopic Roux-en-Y gastric bypass, Metabolic and bariatric surgery accreditation and quality improvement program (MBSAQIP)

## Abstract

**Background:**

The HospitAl stay, Readmission, and Mortality rates (HARM) score is a quality indicator that is easily determined from routine administrative data. However, the HARM score has not yet been applied to patients undergoing bariatric surgery.

**Objective:**

The aims of the present study were to adjust the HARM score to the bariatric population and to validate the ability of the modified HARM score to serve as an inexpensive tool to measure the quality of bariatric surgery.

**Methods:**

A MBSAQIP 2015 PUF database was reviewed. For each discharge, a 1 to 10 score was calculated on the basis of length of stay (LOS), discharge status, and 30-day readmissions. We adjusted the LOS categories to the distribution of LOS in the MBSQIP database. The new LOS categories were used to calculate the modified HARM score, referred to as the BARiatric HARM (BAR-HARM) score. The association between HARM and BAR-HARM scores and complication rate was assessed.

**Results:**

A total of 197,141 cases were evaluated: 98.8% were elective and 1.2% were emergent admissions. The mean individual patient BAR-HARM score was 1.75 ± 1.04 for elective cases, and 2.02 ± 1.45 for emergency cases. The complication rates for the respective BAR-HARM categories ≤ 2, > 2 to 3, > 3 to 4, and > 4 were 3.95, 27.53, 40.14, and 79.97% (*p* < 0.001).

**Conclusions:**

The quality of bariatric surgery can be reliably and validly assessed using the BAR-HARM score, which is a modification of the HARM score.

**Electronic supplementary material:**

The online version of this article (10.1007/s11695-018-3253-5) contains supplementary material, which is available to authorized users.

## Introduction

Evaluation of surgical quality is essential to enable the optimization of patients’ outcomes. Outcome measurement tools are required to ensure high levels of surgical quality; in particular, performance measures are needed for bariatric surgery, which has rapidly increased in popularity. [1, 2] Programs such as the Metabolic and Bariatric Surgery Accreditation and Quality Improvement (MBSAQIP) and the National Surgical Quality Improvement Program (NSQIP) were introduced to standardize processes, identify complications, and improve healthcare quality [3]; however, these programs require dedicated personnel, and are expensive [4].

Keller et al. [5] identified the most important markers of surgical quality as hospital length of stay (LOS), readmission rates, and mortality rates; these variables were integrated into a simple, point-of-care metric referred to as the HospitAl stay, Readmission, and Mortality rates (HARM) score [5]. The HARM score was validated for measuring the quality of care in patients undergoing upper gastrointestinal (GI), hepatobiliary, and colorectal surgery [6]. However, the HARM score has not yet been applied to patients undergoing bariatric surgery.

The aims of the present study were to adjust the HARM score to the bariatric population and to validate the ability of the modified HARM score to serve as an inexpensive tool to measure the quality of bariatric surgery.

## Methods

The present study was based on analyses of data from the MBSAQIP database in the 2015 Participant Use Data File (PUF). MBSAQIP database information is prospectively collected and includes hundreds of standardized and audited demographic variables, preoperative comorbidities, laboratory values, and 30-day postoperative mortality and morbidity outcomes of patients undergoing bariatric procedures in academic and community hospitals in the USA. The PUF contains patient-level data and does not identify hospitals, healthcare providers, or patients. The intended purpose of these files is to provide researchers at participating centers with a data resource that they can analyze to investigate and advance the quality of care delivered to patients undergoing metabolic and bariatric surgery [7, 8]. Metabolic and Bariatric Surgical Clinical Reviewers conduct a data integrity audit of selected participating centers; this process involves the review of multiple charts, some of which are selected randomly and others that are selected based on criteria designed to identify potential reporting errors. The MBSAQIP has determined that the acceptable data integrity audit disagreement rate is 5% or less [7]. All patients with data recorded in the database were eligible for inclusion in the present study, as the MBSAQIP database includes only data from patients who underwent surgical procedures on the esophagus, stomach, and intestines (except the rectum). Records that lacked the necessary data were excluded from the analysis. The procedures were coded using the respective Current Procedural Terminology codes within the range of 43.2xxx–44.6xxx.

The main independent variable was the HARM score (ranging on a scale from 0 to 10), which was calculated as follows: HARM = LOS category (0–5) + discharge status (0/1) × 5 + readmission (0/1). The categorical variables “discharge status” and “readmission” were defined as deceased/alive and yes/no, respectively. If the patient was deceased, the “readmission” variable was taken out of the equation, in accordance with the method described by Keller et al. [5]. LOS was defined as the number of days from the date of initial bariatric or metabolic surgery to the date of hospital discharge and was categorized into the six categories as described in a previous study [5]. Considering that the established LOS categories were defined on the basis of a normal distribution curve for colorectal surgery procedures, we decided to adjust the LOS categories to the distribution of LOS in the MBSQIP database for emergency and elective procedures separately (Table 1). The new LOS categories were used to calculate the modified HARM score, referred to as the BARiatric HARM (BAR-HARM) score, using the following formula: BAR-HARM = modified LOS category (0–5) + discharge status (0/1) × 5 + readmission (0/1). The HARM and the BAR-HARM scores were then categorized into four groups based on score: ≤ 2, > 2 to 3, > 3 to 4, and above 4. The composite endpoint of the complication rate for bariatric surgery was defined by the presence of any of the following conditions: death within 30 days, reoperation within 30 days, readmission within 30 days, transfusion intraoperatively or within the first 72 h postoperatively, superficial incisional surgical site infection, deep surgical site infection, drain still present at 30 days postoperatively, postoperative sepsis, postoperative septic shock, wound disruption, postoperative pneumonia, ventilation required for > 48 h postoperatively, unplanned intubation intra- or postoperatively, postoperative coma for > 24 h, unplanned admission to the intensive care unit within 30 days postoperatively, postoperative pulmonary embolism, postoperative vein thrombosis requiring therapy, postoperative peripheral nerve injury, postoperative urinary tract infection, postoperative progressive renal insufficiency/postoperative acute renal failure requiring dialysis, intra- or postoperative stroke/cerebral vascular accident, and intra- or postoperative myocardial infarction. Complications were classified using the Clavien-Dindo grading system; grade 1 and 2 complications were defined as minor, while grades 3 and 4 were defined as major. The main outcome measures were the assessment of the relationship between the composite HARM/BAR-HARM score and each individual component of the HARM/BAR-HARM score with the complication rates for bariatric surgery.

**Table 1:** Hospital LOS categories by admission type

LOS categories	Based on Keller study		Based on 2015 PUF MBSQIP	
	Emergent (days)	Elective (days)	Emergent (days)	Elective (days)
0	1–5	1–3	< 1	< 1
1	6–8	4	1–< 2	1–< 2
2	9–10	5	2–4	2–< 3
3	11–13	6	5–10	3–< 4
4	14–19	7–8	11–21	4–8
5	+20	+9	+22	+9

***LOS*** length of stay

Statistical analysis was performed using the Pearson chi-squared test to assess associations between categorical variables, while the Student’s *t* test was used for continuous variables. A logistic regression model was used to assess the association between hospital LOS and the presence of complications. Multivariable logistic regression models adjusted for demographics, hospital characteristics, and risk factors were also used to estimate the association between the HARM score/BAR-HARM score categories and complications. The area under the curve (AUC) was calculated to assess the accuracy of both scoring systems [9]. All analyses were performed using SAS® software, University Edition (SAS Institute Inc., Cary, NC, USA).

## Results

### Baseline Characteristics

The present study included a total of 197,141 patients, which constitutes 99.9% of the patients in the 2015 PUF MBSAQIP database. Patients’ demographic data by HARM and BAR-HARM score categories are presented in Supplemental Tables 1 and 2. Of the included patients, 98.8% were elective admissions, and 1.2% were emergency admissions. Mortality rate was 0.13%. Mean patient age was 45.18 ± 12.0 years in the elective group, and 46.6 ± 12.2 years in the emergency group. Mean BMI was 44.8 ± 8.9 kg/m^2^ in the elective group and 35.4 ± 10.0 kg/m^2^ in the emergency group. The mean LOS was 1.9 ± 3.0 days in the elective group and 5.5 ± 11.7 days in the emergency group. Logistical regression analysis revealed that there was an increased risk of developing complications in emergency versus elective surgery (OR 3.59, CI 3.28–3.93).

### HARM Score

The mean individual patient HARM score was 0.20 ± 0.75 in the elective group and 0.70 ± 1.48 in the emergency group. There were increasing odds of overall complications when comparing LOS categories 1 (OR 6.04, CI 5.68–6.42), 2 (OR 9.16, CI 8.365–10.022), 3 (OR 11.039, CI 9.823–12.406), 4 (OR 14.933, CI 13.309–16.755), and 5 (OR 21.529, CI 19.670–23.564) with LOS category 0. Hospital LOS, readmission, and mortality were associated with the presence of complications, providing validation of the HARM score (Table 2). Complication rates were significantly associated with the increasing HARM score. The complication rates for the respective HARM categories ≤ 2, > 2 to 3, > 3 to 4, and > 4 were 8.16, 52.91, 52.36, and 70.25% (*p* < 0.001). Increasing HARM score was associated with increasing adjusted odds ratio for minor complications (AUC 0.525), and major complications (AUC 0.621). The overall HARM score was associated with a greater risk of complications (AUC 0.578, Table 3).

**Table 2:** Complications by HARM score components (LOS categories, readmission, and discharge status)

Complications	Length of stay category* n (%)						Readmission* n (%)		Status* n (%)	
	0	1	2	3	4	5	0	1	0	1
No	171,940 (92.57)	3328 (67.35)	1153 (57.65)	614 (53.02)	544 (45.48)	772 (36.66)	178,351 (95.88)	0 (0.00)	178,351 (90.59)	0 (0.00)
Yes	13,800 (7.43)	1613 (32.65)	847 (42.35)	544 (46.98)	652 (54.52)	1334 (63.34)	7670 (4.12)	10,854 (100.00)	18,524 (9.41)	269 (100.0)

*n* number of patients

*χ^2^ test

**Table 3:** Odds ratio and 95% confidence interval for complication for HARM score, risk adjusted

HARM score	Odds ratio	95% confidence interval	
All complications			
≤ 2	Ref.		
> 2–3	12.645	11.328	14.115
> 3–4	12.369	11.000	13.908
> 4	26.571	24.388	28.949
CD grade 1/2			
≤ 2	Ref.		
> 2–3	5.068	4.409	5.826
> 3–4	3.796	3.224	4.469
> 4	2.408	2.116	2.740
CD grade 3/4			
≤ 2	Ref.		
> 2–3	13.323	11.849	14.981
> 3–4	15.561	13.769	17.586
> 4	39.505	36.392	42.884

*CD* Clavien-Dindo classification, *HARM* HospitAl length of stay, Readmission, and Mortality

### BAR-HARM Score

The mean individual patient BAR-HARM score was 1.75 ± 1.04 for elective cases and 2.02 ± 1.45 for emergency cases. There were increasing odds of overall complications when comparing modified LOS categories 1 (OR 1.062, CI 0.980–1.151), 2 (OR 1.705, CI 1.577–1.843), 3 (OR 4.129, CI 3.799–4.488), 4 (OR 12.643, CI 12.643–13.764), and 5 (OR 38.176, CI 30.308–38.176) with LOS category 0. Modified categories of the hospital LOS and other components of the BAR-HARM score were associated with the presence of complications, validating the proposed scoring system (Table 4). Complication rates were significantly associated with an increasing BAR-HARM score. The complication rates for the respective BAR-HARM categories ≤ 2, > 2 to 3, > 3 to 4, and > 4 were 3.95, 27.53, 40.14, and 79.97% (*p* < 0.001). Increasing BAR-HARM score was associated with an increasing adjusted odds ratio for minor complications (AUC 0.730) and for major complications (AUC 0.803). The overall BAR-HARM score was directly and strongly correlated with a greater risk of complications (AUC 0.781, Table 5).

**Table 4:** Complications by BAR-HARM score components (modified LOS categories, readmission, and discharge status)

Complications	Modified length of stay category* n (%)						Readmission* n (%)		Status* n (%)	
	0	1	2	3	4	5	0	1	0	1
No	14,654 (95.17)	67,877 (94.89)	74,938 (92.04)	14,607 (82.69)	5505 (60.94)	770 (36.70)	178,351 (95.88)	0 (0.00)	178,351 (90.59)	0 (0.00)
Yes	743 (4.83)	3655 (5.11)	6477 (7.96)	3058 (17.31)	3529 (39.06)	1328 (63.30)	7670 (4.12)	10,854 (100.00)	18,524 (9.41)	269 (100.0)

*n* number of patients

*χ^2^ test

**Table 5:** Odds ratio and 95% confidence interval for complication for BAR-HARM score, risk adjusted

BAR-HARM score	Odds ratio	95% confidence interval	
All complications			
≤ 2	Ref.		
> 2–3	9.240	8.881	9.614
> 3–4	16.308	15.535	17.119
> 4	97.082	89.368	105.463
CD grade 1/2			
≤ 2	Ref.		
> 2–3	7.921	7.534	8.328
> 3–4	9.618	9.038	10.234
> 4	11.279	10.365	12.273
CD grade 3/4			
≤ 2	Ref.		
> 2–3	7.978	7.538	8.444
> 3–4	16.382	15.396	17.432
> 4	84.887	78.790	91.455

*CD* Clavien-Dindo classification, *BAR-HARM* Bariatric-HospitAl length of stay, Readmission, and Mortality

## Discussion

To the best of our knowledge, the present study is the first to validate the ability of the HARM score to serve as a performance measure in patients undergoing bariatric surgery. We assessed the HARM score using the Clavien-Dindo classification system and showed that the risk of major complications increased with increasing HARM score. However, the HARM score was originally developed based on data derived from colorectal surgical procedures. The accuracy of the HARM score in detecting complications in bariatric patients was poor in our study. The majority of bariatric patients in this analysis were in the HARM score < 2 category (97.44%); in contrast, Crawshaw et al. [6] reported that the HARM score < 2 category comprised only 49% of a mixed group of upper GI, hepatobiliary, and colorectal surgery patients. As bariatric surgery is associated with a shorter LOS and lower complication rates in comparison with colorectal surgery, we decided to adjust the LOS categories to suit the bariatric population on the basis of the MBSQIP database [10]. The modified HARM score, referred to as the BAR-HARM score, had good accuracy in detecting complications in the bariatric population. The present results show that this modified BAR-HARM score has great potential for application as a simple quality indicator for bariatric surgery.

Bariatric surgery is a dynamically developing area of surgical practice. Considering the wide range of procedures offered by providers, there is a high variance in practices among bariatric surgeons. The monitoring of outcomes based on quality indicators enables the identification and recognition of positive variance, as well as the early identification of poorly performing centers.

In 2006, Maagard et al. [11] reported the first formal attempt to develop quality indicators for bariatric surgery and proposed 51 indicators rated as valid measures of good quality care for bariatric surgery. The large number of indicators made the proposed system difficult to use for benchmarking. Programs like the MBSAQIP and the NSQIP were introduced to standardize processes, identify complications, and improve healthcare quality [3]; however, these programs require dedicated personnel and are expensive [4].

The HARM score and the BAR-HARM score are quality indicators that are easily determined from routine administrative data. The scores differ from other performance measures used to describe outcomes and quality of surgery and can be calculated using available administrative data, without the additional cost of software or ongoing costs for maintenance or personnel. As there is no investment cost, Keller et al. [5] reported that the HARM score may decrease administrative costs associated with quality care improvement programs. Hence, the BAR-HARM score may be a good alternative to expensive programs like the MBSAQIP, which are used to measure outcomes in surgery. Previous research has proven the strength and validity of the HARM score and its individual components in patients undergoing upper GI, hepatobiliary, and colorectal surgery [6]. Hospital LOS, readmission, and mortality directly affect patient outcomes and are closely related to quality measurements [12–14]. Mortality may be the main short-term outcome of interest when comparing surgical performance across hospitals [15]. Postoperative complications influence the components of the HARM score and the BAR-HARM score and will result in prolonged LOS or readmission. Thus, an increased HARM score and BAR-HARM score reflects increased complication rates, without the need for detailed review of medical records.

The present study had some limitations that must be considered. First, the MBSAQIP database is a large, observational database, and it may contain errors or omissions that could distort or alter our findings. Second, data enabling center identification are not available in the MBSAQIP 2015 PUF. Thus, we were not able to assess whether hospitals with different HARM and BAR-HARM scores have different complication rates. However, this association was reported in GI surgery by Crawshaw et al. [6].

## Conclusion

The quality of bariatric surgery can be reliably and validly assessed using the BAR-HARM score, which is a modification of the HARM score. The three components of the BAR-HARM score (LOS, readmissions, and mortality) are strongly associated with complications, and the BAR-HARM score has significant value as a tool with which to discriminate the quality of care. The BAR-HARM score can be easily calculated from available administrative datasets without the need for additional costs.

## Electronic Supplementary Material

ESM 1 (DOCX 92.8 kb)

ESM 2 (DOCX 94.4 kb)
