# Supplementary material for: Application of HARM Score to Measure Surgical Quality and Outcomes in Bariatric Patients
Source: Obes Surg. 2018 Apr 27;28(9):2815–9. doi: 10.1007/s11695-018-3253-5 (PMC6132742; doi:10.1007/s11695-018-3253-5)
Supplement: Supplementary file 1 — (DOCX 92.8 kb) [file 11695_2018_3253_MOESM1_ESM.docx]

**Supplement Table 1. Description of study population categorized using HARM score.**

| **Variable** | **HARM<=2**  N=192 100  (97.44%) | | **HARM>2-3**  N=1304  (0.66%) | | **HARM >3-4**  N=1142  (0.58%) | | **HARM >4**  N= 2595  (1.32%) | | **p-value** |
| --- | --- | --- | --- | --- | --- | --- | --- | --- | --- |
|  | *Mean* | *SD* | *Mean* | *SD* | *Mean* | *SD* | *Mean* | *SD* |  |
| **Age** | 45.08 | ±11.98 | 48.98 | ±11.77 | 50.10 | ±11.68 | 49.65 | ±12.25 | <0.001* |
| **BMI** | 44.75 | ±8.82 | 44.18 | ±11.92 | 43.02 | ±12.50 | 43.82 | ±12.47 | <0.001* |
| **LOS** | 1.64 | ±0.94 | 5.95 | ±1.22 | 7.50 | ±1.77 | 20.30 | ±19.11 | <0.001* |
|  | *n* | *%* | *n* | *%* | *n* | *%* | *n* | *%* |  |
| **Sex (Female)** | 153627 | 79.97% | 1039 | 79.68% | 897 | 78.55% | 1980 | 76.30% | <0.001** |
| **Race (white)** | 143553 | 74.7% | 962 | 73.7% | 844 | 73.9% | 1839 | 70.87% | <0.001** |
| **Hypertension** | 92176 | 47.98% | 723 | 55.44% | 650 | 56.92% | 1423 | 54.84% | <0.001** |
| **Diabetes type 2** | 47826 | 24.90% | 414 | 31.75% | 368 | 32.22% | 780 | 30.06% | <0.001** |
| **Hyperlipidemia** | 46539 | 24.23% | 409 | 31.37% | 359 | 31.44% | 759 | 29.25% | <0.001** |
| **Sleep Apnea** | 66542 | 34.64% | 542 | 41.56% | 430 | 37.65% | 968 | 37.30% | <0.001** |
| **GERD** | 62272 | 32.42% | 567 | 43.48% | 505 | 44.22% | 1088 | 41.93% | <0.001** |
| **Smoker** | 17094 | 8.90% | 129 | 9.89% | 126 | 11.03% | 263 | 10.13% | <0.001** |
| **Chronic steroids use** | 2975 | 1.55% | 44 | 3.37% | 34 | 2.98% | 97 | 3.74% | <0.001** |
| **Elective cases** | \|  \| 189939 \| \| --- \| --- \| | 98.88% | 1253 | 96.0% | 1091 | 95.5% | 2432 | 93.72% | <0.001** |

HARM - HospitAl length of stay, Readmissions and Mortality**,** LOS - length of stay,

* - Test Kruskala-Wallisa, ****-**X^2^ test
