# Supplementary material for: Application of HARM Score to Measure Surgical Quality and Outcomes in Bariatric Patients
Source: Obes Surg. 2018 Apr 27;28(9):2815–9. doi: 10.1007/s11695-018-3253-5 (PMC6132742; doi:10.1007/s11695-018-3253-5)
Supplement: Supplementary file 2 — (DOCX 94.4 kb) [file 11695_2018_3253_MOESM2_ESM.docx]

**Supplement Table 2. Description of study population categorized using BAR-HARM score.**

| **Variable** | **BAR-HARM<=2**  N=163 944  (83.16%) | | **BAR-HARM>2-3**  N= 20 157  (10.22%) | | **BAR-HARM >3-4**  N= 9 196  (4.66%) | | **BAR-HARM >4**  N= 3 844  (1.95%) | | **p-value** |
| --- | --- | --- | --- | --- | --- | --- | --- | --- | --- |
|  | *Mean* | *SD* | *Mean* | *SD* | *Mean* | *SD* | *Mean* | *SD* |  |
| **Age** | 44.86 | ±11.93 | 45.82 | ±12.16 | 48.21 | ±12.19 | 48.90 | ±12.09 | <0.001* |
| **BMI** | 44.63 | ±8.59 | 45.61 | ±9.72 | 44.65 | ±10.97 | 44.12 | ±12.28 | <0.001* |
| **LOS** | 1.38 | ±0.66 | 2.85 | ±0.64 | 4.66 | ±1.64 | 15.17 | ±17.43 | <0.001* |
|  | *n* | *%* | *n* | *%* | *n* | *%* | *n* | *%* |  |
| **Sex (Female)** | 130531 | 79.6% | 16630 | 82.5% | 7370 | 80.1% | 3012 | 78.36% | <0.001** |
| **Race (white)** | 123356 | 75.24% | 14425 | 71.56% | 6688 | 72.73% | 2729 | 70.99% | <0.001** |
| **Hypertension** | 77155 | 47.06% | 10612 | 52.65% | 5090 | 55.35% | 2115 | 55.02% | <0.001** |
| **Diabetes type 2** | 39269 | 23.95% | 5967 | 29.6% | 2972 | 32.32% | 1180 | 30.70% | <0.001** |
| **Hyperlipidemia** | 38771 | 23.65% | 5448 | 27.03% | 2719 | 29.57% | 1128 | 29.34% | <0.001** |
| **Sleep Apnea** | 55422 | 33.81% | 7804 | 38.72% | 3764 | 40.93% | 1492 | 38.81% | <0.001** |
| **GERD** | 51318 | 31.30% | 7425 | 36.84% | 3962 | 43.08% | 1727 | 44.93% | <0.001** |
| **Smoker** | 14416 | 8.79% | 1852 | 9.19% | 949 | 10.32% | 395 | 10.28% | <0.001** |
| **Chronic steroids use** | 2387 | 1.46% | 374 | 1.86% | 257 | 2.79% | 132 | 3.43% | <0.001** |
| **Elective cases** | 162293 | 98.99% | 19735 | 97.91% | 9006 | 97.93% | 3681 | 95.76% | <0.001** |

HARM - HospitAl length of stay, Readmissions and Mortality**,** LOS - length of stay,

* - Test Kruskala-Wallisa, ****-**X^2^ test
